# Supplementary material for: Assembly and Analysis of the Complete Mitochondrial Genome of Capsella bursa-pastoris
Source: Plants (Basel). 2020 Apr 8;9(4):469. doi: 10.3390/plants9040469 (PMC7238199; doi:10.3390/plants9040469)
Supplement: Supplementary file 1 [file plants-09-00469-s001.pdf]

plants

MDPI

Supplementary

Table S1. Repeats in the *C. bursa-pastoris* mitogenome

Bit score	Repeat ID	Alignment length (bp)	Identity (%)	First repeat unit position	Second repeat unit position	Repeat type
1528	Rep_1	854	98.9	54,219-55,072	49,506-48,653	inverted
1173	Rep_2	635	100.0	158,147-158,781	106,515-105,881	inverted
987	Rep_3	538	99.8	134,019-134,556	116,143-116,679	direct
843	Rep_4	626	91.1	153,833-154,456	107,131-106,508	inverted
835	Rep_5	493	97.4	281,667-282,157	48,414-47,924	inverted
833	Rep_6	546	94.7	251,830-252,373	287,008-287,539	direct
767	Rep_7	418	99.8	220,620-221,037	54,655-55,072	direct
749	Rep_8	456	96.7	274,872-275,317	236,062-235,608	inverted
739	Rep_9	404	99.8	238,171-238,573	17,371-17,774	direct
726	Rep_10	420	97.9	220,623-221,042	49,067-48,648	inverted
658	Rep_11	356	100.0	64,729-65,084	26,047-26,402	direct
604	Rep_12	327	100.0	93,763-94,089	62,446-62,772	direct
601	Rep_13	341	98.5	215,628-215,968	49,513-49,174	inverted
588	Rep_14	334	98.5	215,635-215,968	54,219-54,551	direct
538	Rep_15	297	99.3	280,911-281,207	199,757-200,053	direct
444	Rep_16	249	98.8	63,554-63,802	42,130-41,882	inverted
444	Rep_17	249	98.8	271,231-271,479	154,362-154,610	direct
425	Rep_18	230	100.0	193,196-193,425	18,618-18,847	direct
407	Rep_19	254	95.7	167,404-167,655	64,672-64,419	inverted
359	Rep_20	254	92.1	281,849-282,102	119,998-120,251	direct
351	Rep_21	209	97.1	192,505-192,713	43,599-43,392	inverted
351	Rep_22	209	97.1	43,392-43,599	192,713-192,505	inverted
300	Rep_23	252	88.1	119,998-120,249	48,232-47,981	inverted
279	Rep_24	283	84.8	281,467-281,748	119,542-119,819	direct
268	Rep_25	158	97.5	174,149-174,306	43,547-43,392	inverted
252	Rep_26	136	100.0	89,791-89,926	89,762-89,897	direct
246	Rep_27	147	97.3	235,325-235,471	42,993-42,850	inverted
246	Rep_28	168	93.5	174,149-174,314	192,557-192,722	direct
220	Rep_29	122	99.2	15,834-15,955	4,835-4,956	direct
211	Rep_30	117	99.1	17,777-17,893	6,904-6,788	inverted
207	Rep_31	112	100.0	154,513-154,624	80,756-80,867	direct
204	Rep_32	113	99.1	235,991-236,103	154,656-154,544	inverted
198	Rep_33	107	100.0	89,820-89,926	89,762-89,868	direct
193	Rep_34	104	100.0	282,199-282,302	41,386-41,283	inverted
193	Rep_35	121	95.9	176,439-176,556	195,247-195,367	direct
182	Rep_36	98	100.0	271,382-271,479	80,756-80,853	direct
165	Rep_37	95	97.9	271,231-271,325	106,602-106,508	inverted
161	Rep_38	94	97.9	218,934-219,026	68,248-68,155	inverted
156	Rep_39	84	100.0	4,806-4,889	4,775-4,858	direct

150	Rep_40	93	95.7	281,281-281,373	64,419-64,511	direct
150	Rep_41	81	100.0	80,787-80,867	236,103-236,023	inverted
148	Rep_42	120	89.2	181,213-181,332	171,539-171,422	inverted
145	Rep_43	90	95.6	231,258-231,347	22,139-22,228	direct
145	Rep_44	78	100.0	89,762-89,839	89,849-89,926	direct
141	Rep_45	207	80.2	167,083-167,282	31,198-31,395	direct
141	Rep_46	82	97.6	48,333-48,414	119,819-119,738	inverted
135	Rep_47	88	94.3	282,108-282,195	217,652-217,739	direct
134	Rep_48	78	97.4	125,984-126,061	31,420-31,497	direct
124	Rep_49	67	100.0	271,413-271,479	236,103-236,037	inverted
122	Rep_50	72	97.2	213,800-213,871	36,349-36,420	direct
121	Rep_51	69	98.6	235,499-235,566	287,208-287,140	inverted
121	Rep_52	69	98.6	287,140-287,208	235,566-235,499	inverted
111	Rep_53	69	95.7	186,245-186,313	60,759-60,827	direct
111	Rep_54	63	98.4	73,738-73,800	177,779-177,841	direct
110	Rep_55	93	88.2	167,164-167,255	120,504-120,413	inverted
108	Rep_56	69	95.7	134,445-134,510	49,164-49,096	inverted
108	Rep_57	69	95.7	116,569-116,634	54,561-54,629	direct
108	Rep_58	69	95.7	54,561-54,629	116,569-116,634	direct
108	Rep_59	69	95.7	54,561-54,629	134,445-134,510	direct
106	Rep_60	57	100.0	37,711-37,767	4,923-4,867	inverted
106	Rep_61	57	100.0	15,866-15,922	37,767-37,711	inverted
106	Rep_62	63	96.8	237,791-237,853	234,695-234,757	direct
104	Rep_63	62	96.8	157,646-157,707	30,507-30,568	direct
104	Rep_64	62	96.8	30,507-30,568	157,646-157,707	direct
100	Rep_65	64	95.3	169,718-169,780	15,912-15,975	direct
100	Rep_66	54	100.0	276,261-276,314	212,291-212,238	inverted
99	Rep_67	53	100.0	4,837-4,889	4,775-4,827	direct
99	Rep_68	71	91.5	55,089-55,159	31,151-31,081	inverted
99	Rep_69	66	93.9	84,339-84,404	84,306-84,370	direct
97.1	Rep_70	92	87.0	215,611-215,700	287,569-287,483	inverted
97.1	Rep_71	55	98.2	4,804-4,858	15,834-15,888	direct
97.1	Rep_72	52	100.0	139,967-140,018	116,867-116,816	inverted
97.1	Rep_73	52	100.0	116,816-116,867	140,018-139,967	inverted
95.3	Rep_74	51	100.0	113,885-113,935	100,171-100,121	inverted
93.5	Rep_75	53	98.1	15,836-15,888	4,775-4,827	direct
93.5	Rep_76	53	98.1	167,302-167,354	119,248-119,196	inverted
91.6	Rep_77	49	100.0	127,697-127,745	9,777-9,825	direct
91.6	Rep_78	49	100.0	89,762-89,810	89,878-89,926	direct
91.6	Rep_79	49	100.0	9,777-9,825	127,697-127,745	direct
89.8	Rep_80	72	88.9	49,823-49,894	427-498	direct
89.8	Rep_81	58	94.8	217,098-217,154	127,197-127,140	inverted
86.1	Rep_82	59	93.2	236,033-236,091	11,203-11,146	inverted
86.1	Rep_83	59	93.2	154,556-154,614	11,146-11,203	direct
86.1	Rep_84	49	98.0	65,516-65,564	65,488-65,536	direct
86.1	Rep_85	59	93.2	11,146-11,203	80,799-80,857	direct
86.1	Rep_86	59	93.2	11,146-11,203	236,091-236,033	inverted

82.4	Rep_87	44	100.0	157,293-157,336	32,088-32,045	inverted
80.5	Rep_88	46	97.8	169,718-169,763	4,913-4,958	direct
76.8	Rep_89	58	91.4	49,441-49,496	287,483-287,539	direct
76.8	Rep_90	56	91.1	120,413-120,468	31,369-31,314	inverted
76.8	Rep_91	58	91.4	287,483-287,539	54,284-54,229	inverted
76.8	Rep_92	41	100.0	270,534-270,574	129,190-129,150	inverted
73.1	Rep_93	39	100.0	63,799-63,837	42,987-42,949	inverted
73.1	Rep_94	39	100.0	235,331-235,369	63,799-63,837	direct
73.1	Rep_95	86	82.6	56,025-56,109	152,824-152,742	inverted
71.3	Rep_96	38	100.0	129,195-129,232	49,268-49,305	direct
71.3	Rep_97	38	100.0	104,080-104,117	74,139-74,176	direct
71.3	Rep_98	38	100.0	49,268-49,305	129,195-129,232	direct
71.3	Rep_99	38	100.0	54,420-54,457	129,232-129,195	inverted
71.3	Rep_100	38	100.0	129,195-129,232	215,874-215,837	inverted
71.3	Rep_101	41	97.6	222,241-222,281	227,867-227,907	direct
67.6	Rep_102	40	97.5	158,734-158,773	17,260-17,298	direct
67.6	Rep_103	36	100.0	274,872-274,907	80,828-80,863	direct
67.6	Rep_104	36	100.0	237,815-237,850	151,796-151,761	inverted
67.6	Rep_105	36	100.0	230,870-230,905	163,266-163,231	inverted
67.6	Rep_106	36	100.0	151,761-151,796	237,850-237,815	inverted
65.8	Rep_107	35	100.0	47,893-47,927	41,183-41,217	direct
65.8	Rep_108	35	100.0	171,055-171,089	76,111-76,077	inverted
62.1	Rep_109	40	95.0	36,396-36,435	4,824-4,787	inverted
62.1	Rep_110	40	95.0	36,396-36,435	4,886-4,849	inverted
62.1	Rep_111	40	95.0	4,849-4,886	36,435-36,396	inverted
62.1	Rep_112	40	95.0	4,787-4,824	36,435-36,396	inverted
62.1	Rep_113	33	100.0	101,718-101,750	101,764-101,796	direct
60.2	Rep_114	39	94.9	113,939-113,977	13,248-13,285	direct
60.2	Rep_115	39	94.9	13,248-13,285	113,939-113,977	direct
60.2	Rep_116	32	100.0	132,583-132,614	124,779-124,810	direct
58.4	Rep_117	38	94.7	163,229-163,266	15,817-15,853	direct
58.4	Rep_118	31	100.0	55,439-55,469	55,490-55,520	direct
58.4	Rep_119	31	100.0	235,303-235,333	87,622-87,652	direct
58.4	Rep_120	34	97.1	229,927-229,960	89,804-89,837	direct
58.4	Rep_121	34	97.1	229,927-229,960	89,862-89,895	direct
58.4	Rep_122	44	90.9	125,370-125,413	111,823-111,782	inverted
58.4	Rep_123	38	94.7	15,817-15,853	163,229-163,266	direct
58.4	Rep_124	31	100.0	199,776-199,806	167,953-167,923	inverted
58.4	Rep_125	34	97.1	89,775-89,808	229,927-229,960	direct
58.4	Rep_126	34	97.1	89,833-89,866	229,927-229,960	direct
58.4	Rep_127	34	97.1	89,891-89,924	229,927-229,960	direct
58.4	Rep_128	31	100.0	78,644-78,674	239,736-239,706	inverted
58.4	Rep_129	34	97.1	243,574-243,607	244,392-244,425	direct
56.5	Rep_130	30	100.0	204,234-204,263	74,142-74,171	direct
56.5	Rep_131	30	100.0	74,142-74,171	204,234-204,263	direct
54.7	Rep_132	36	94.4	230,870-230,905	15,853-15,819	inverted
54.7	Rep_133	43	90.7	64,690-64,728	31,369-31,327	inverted

54.7	Rep_134	49	87.8	167,213-167,261	64,728-64,684	inverted
54.7	Rep_135	29	100.0	124,675-124,703	96,424-96,396	inverted
54.7	Rep_136	29	100.0	96,396-96,424	124,703-124,675	inverted
54.7	Rep_137	43	90.7	64,690-64,728	167,255-167,213	inverted
54.7	Rep_138	36	94.4	15,819-15,853	230,905-230,870	inverted
54.7	Rep_139	29	100.0	158,740-158,768	285,725-285,697	inverted
52.8	Rep_140	28	100.0	121,426-121,453	62,607-62,580	inverted
52.8	Rep_141	28	100.0	93,897-93,924	121,453-121,426	inverted

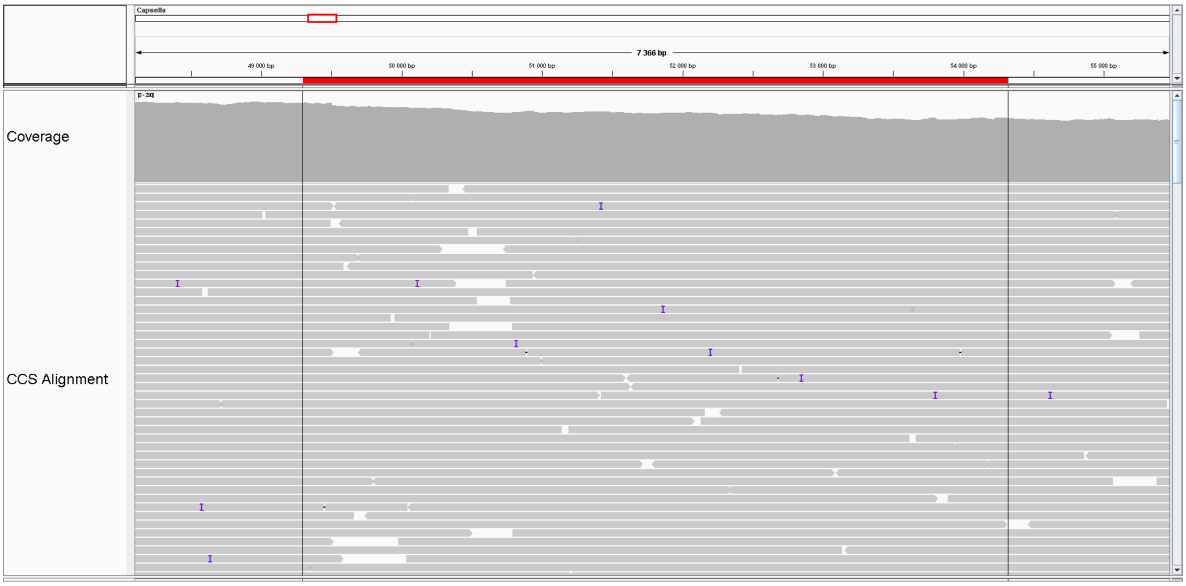
Capsella
7 366 bp
49 000 bp
50 000 bp
51 000 bp
52 000 bp
53 000 bp
54 000 bp
55 000 bp
Coverage
CCS Alignment

(a)

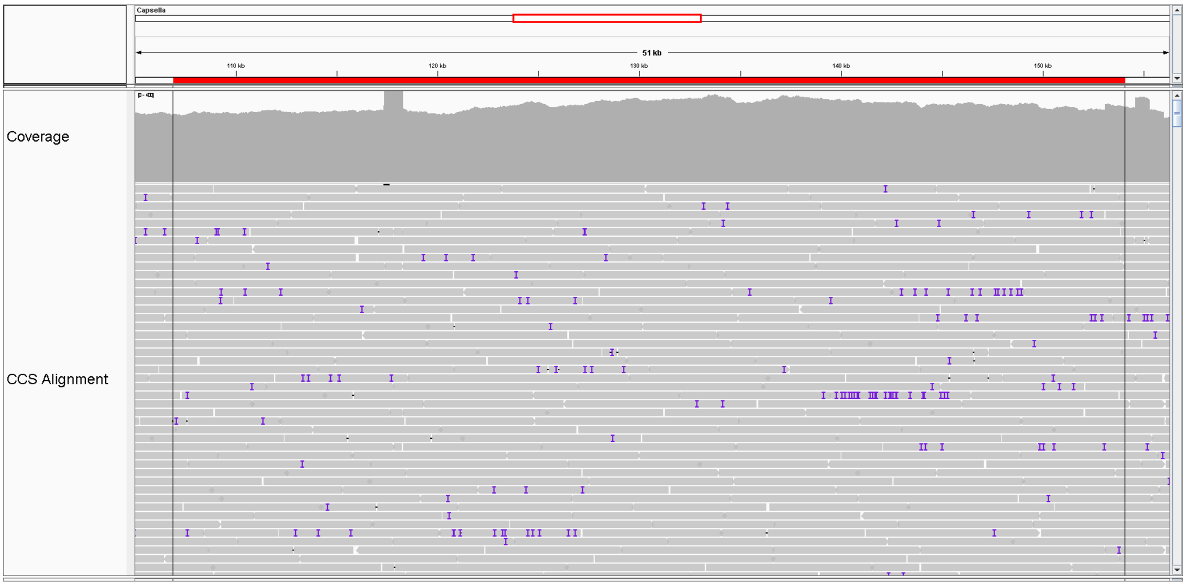
Capsella
51 kb
110 kb
120 kb
130 kb
140 kb
150 kb
Coverage
CCS Alignment

(b)

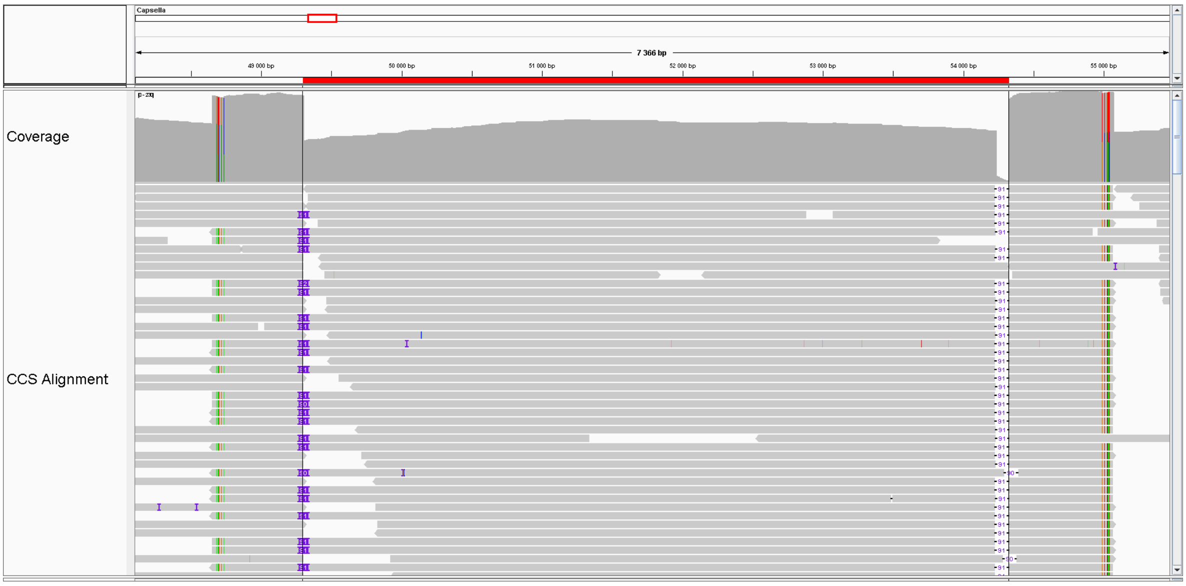
Capsella
7 366 bp
49 000 bp
50 000 bp
51 000 bp
52 000 bp
53 000 bp
54 000 bp
55 000 bp
Coverage
CCS Alignment

(c)

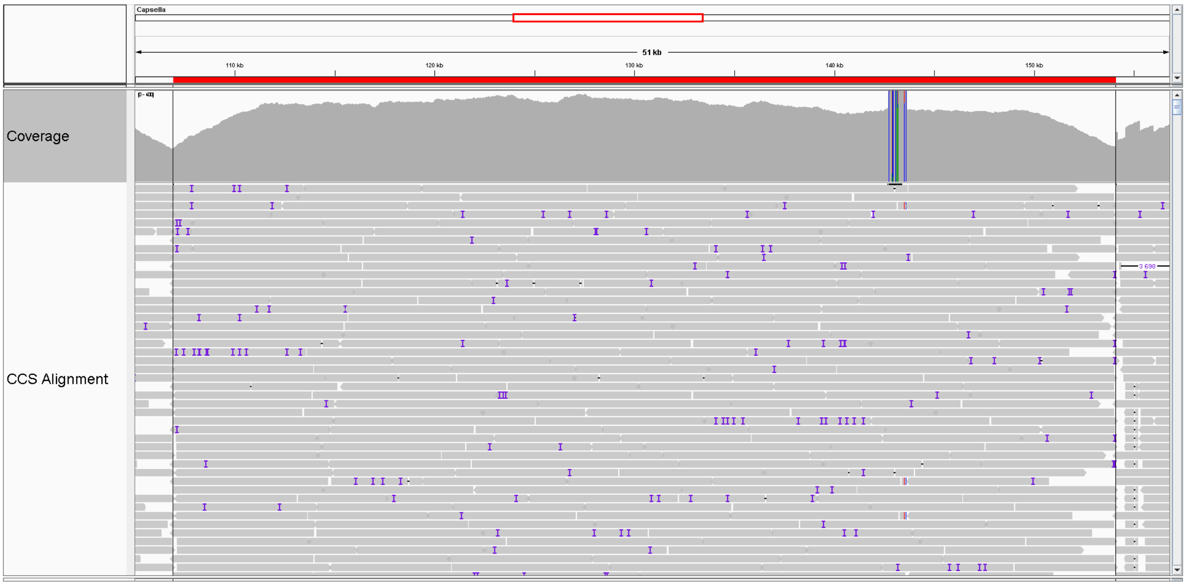
Capsella
51 kb
110 kb
120 kb
130 kb
140 kb
150 kb
Coverage
CCS Alignment

(d)

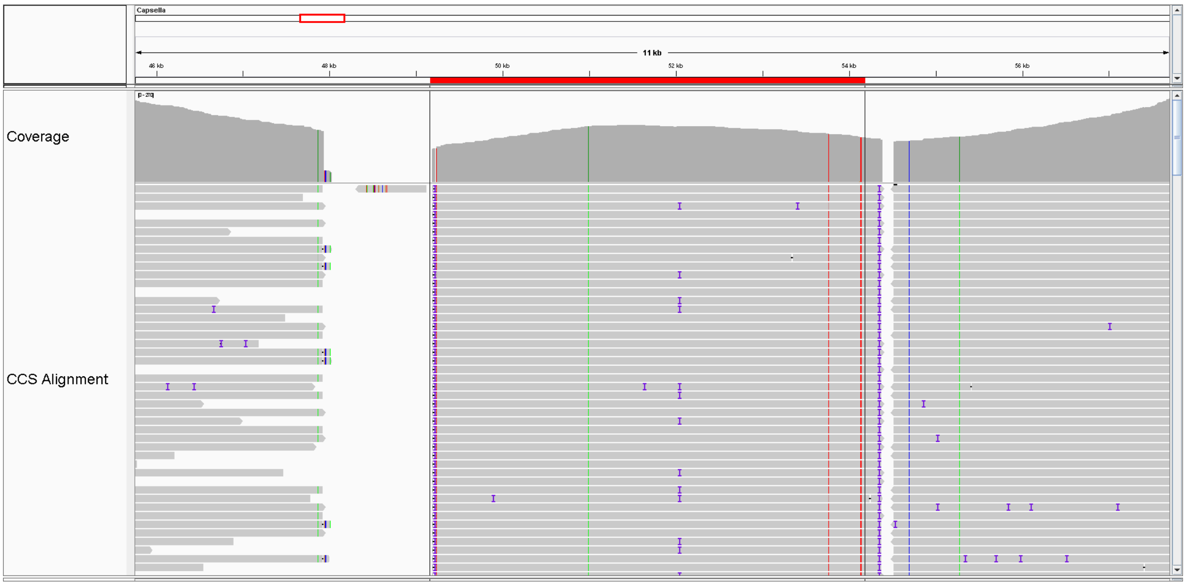
Capsella
11 kb
46 kb
48 kb
50 kb
52 kb
54 kb
56 kb
Coverage
CCS Alignment

(e)

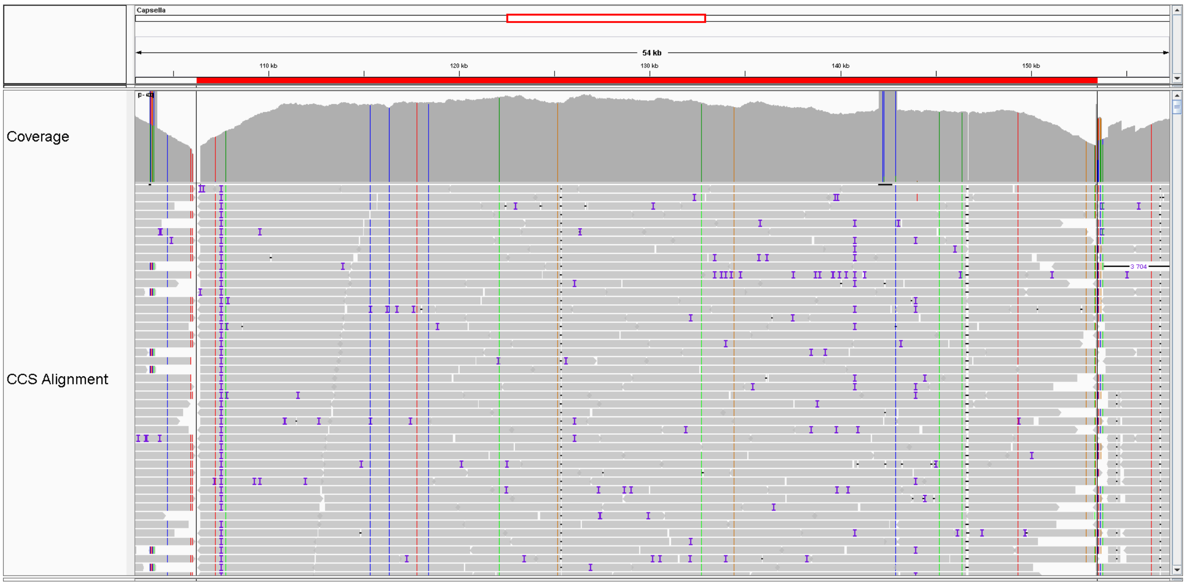

(f)

Figure S1. Alignment of all CCS reads to the *C. bursa-pastoris* and *C. rubella* mtDNA sequences. Alignment map of the CCS reads to the originally assembled *C. bursa-pastoris* mtDNA in ~5 kbp inversion region (**a**) and ~47 kbp inversion region (**b**); to the *C .bursa-pastoris* mtDNA with manually inverted regions in ~5 kbp inversion region (**c**) and ~47 kbp inversion region (**d**); to the *C. rubella* mtDNA in ~5 kbp inversion region (**e**) and ~47 kbp inversion region (**f**). Red region on the genome coordinate axis represents inversion with borders as vertical black lines across the alignment. Grey histogram track represents read coverage, and horizontal bars represent reads aligned to the reference. The image has been created in IGV browser software with the default color legends.

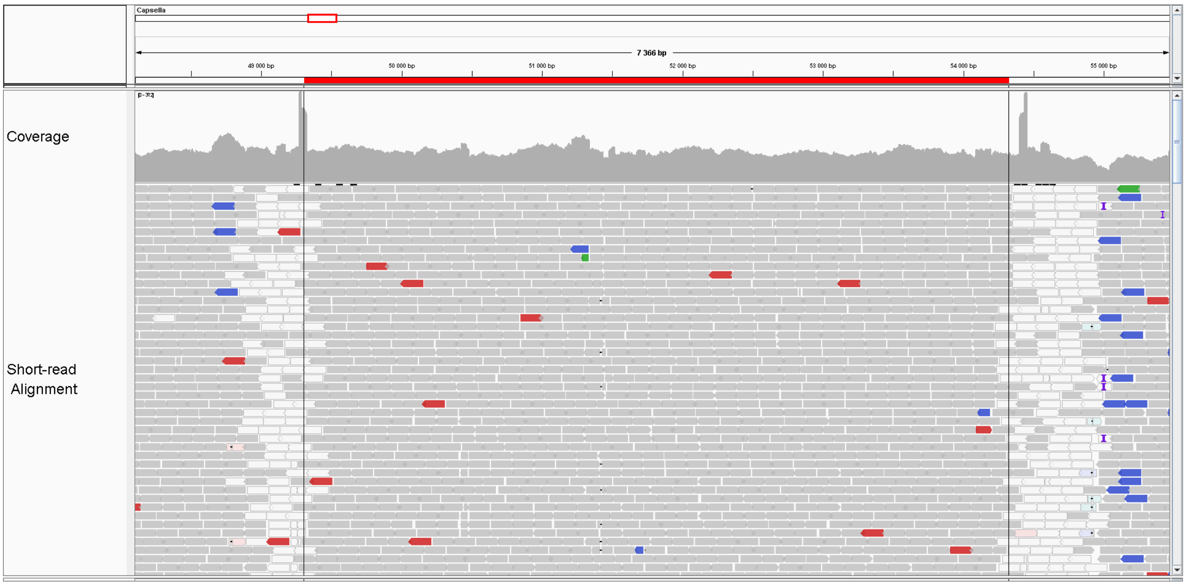
Capsella
7 366 bp
49 000 bp
50 000 bp
51 000 bp
52 000 bp
53 000 bp
54 000 bp
55 000 bp
Coverage
Short-read Alignment

(a)

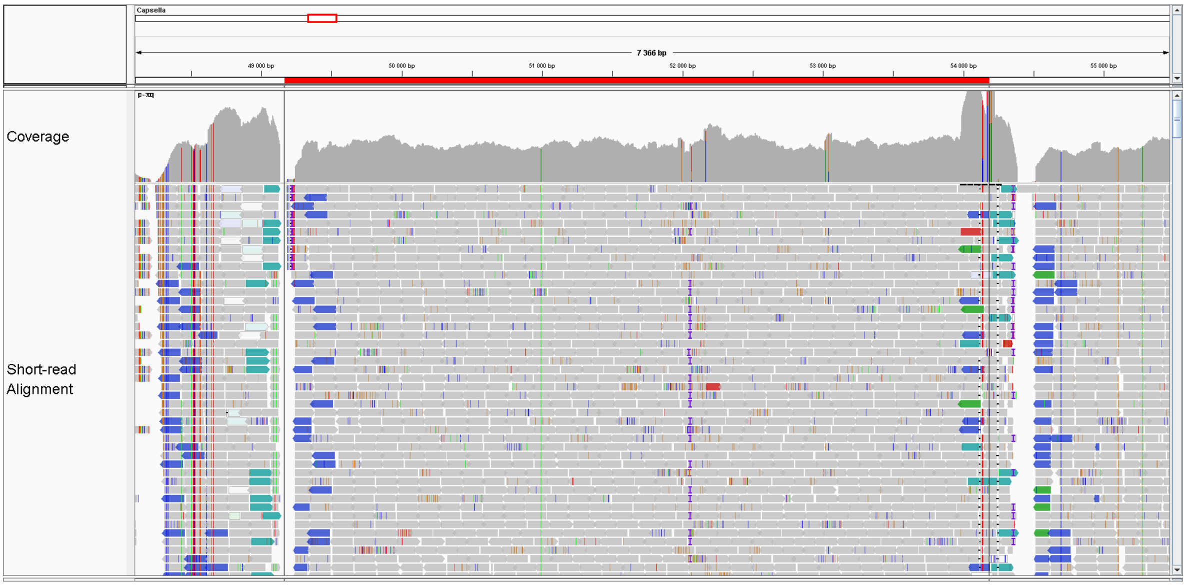
Capsella
7 366 bp
49 000 bp
50 000 bp
51 000 bp
52 000 bp
53 000 bp
54 000 bp
55 000 bp
Coverage
Short-read Alignment

(b)

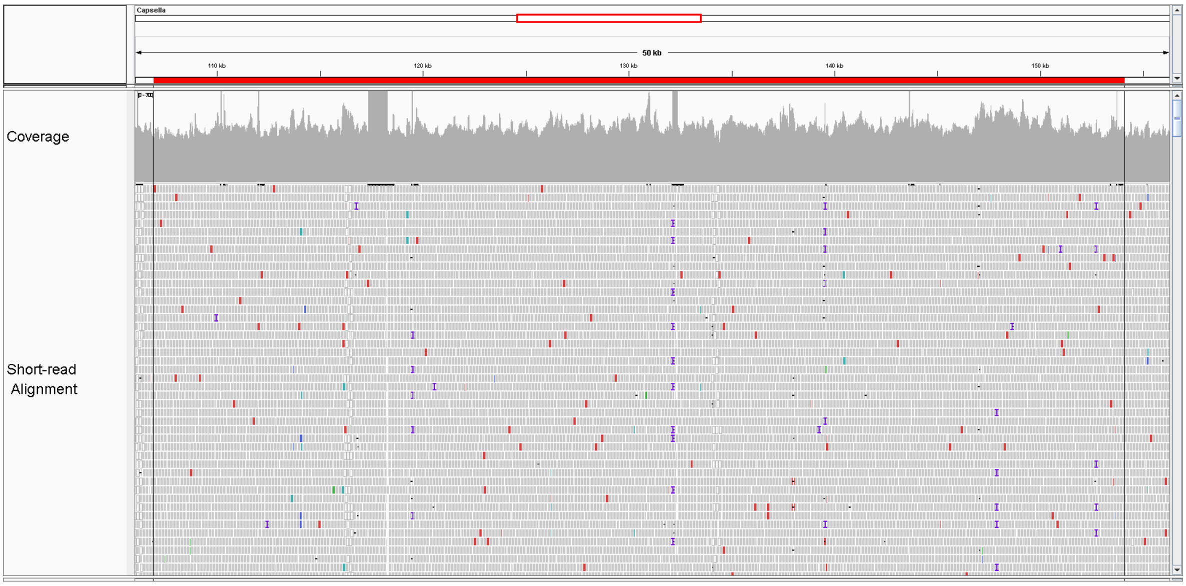

(c)

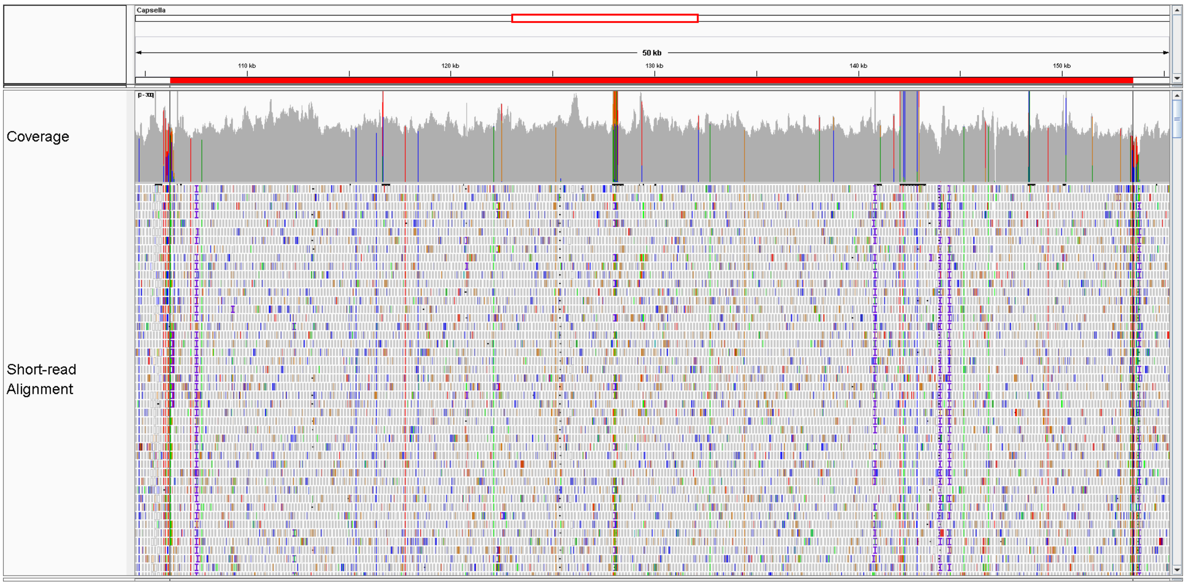

(d)

Figure S2. Alignment of the *C. orientalis* Illumina pair-end reads to the *C. bursa-pastoris* and *C. rubella* mtDNA sequences. Short-read alignment to *C. bursa-pastoris* (**a**) and *C. rubella* (**b**) in ~5 kbp inversion region; Short-read alignment to *C. bursa-pastoris* (**c**) and *C. rubella* (**d**) in ~47 kbp inversion region. Red region on the genome coordinate axis represents inversion with borders as vertical black lines across the alignment. Grey histogram track represents read coverage, and horizontal bars represent reads aligned to the reference. The image has been created in IGV browser software with the default color legends.

Table S2. Comparison of the gene content between *C. bursa-pastoris*, *C. rubella,* and *A. thaliana* mitogenomes.

Gene group	Gene	*C. bursa-pastoris*		*C. rubella*		*A. thaliana*	
		length, bp	Exons	length, bp	Exons	length, bp	Exons
ATP synthase	*atp1*	1524	1	1524	1	1524	1
	atp4	579	1	579	1	579	1
	atp6	1128	1	1128	1	1158	1
	atp6_2	–	–	–	–	1050	1
	atp8	477	1	477	1	477	1
	atp9	225	1	258	1	225	1
Cytochrome c biogenesis	*ccmB*	621	1	621	1	621	1
	ccmC	771	1	771	1	771	1
	ccmFC	1329	2	1359	2	1329	2
	ccmFN1	1143	1	1143	1	1149	1
	ccmFN2	612	1	612	1	612	1
Ubiquinol-cytochrome c reductase	*cob*	1182	1	1182	1	1182	1
Cytochrome c oxidase	*cox1*	1584	1	1584	1	1584	1
	cox2	783	2	783	2	783	2
	cox3	798	1	798	1	798	1
Maturase	*matR*	1971	1	2031	1	1971	1
Membrane transport protein	*mttB*	834	1	834	1	843	1
NADH dehydrogenase	*nad1*	978	5	978	5	978	5
	nad2	1467	5	1467	5	1467	5
	nad3	357	1	357	1	357	1
	nad4	1488	4	1488	4	1488	4
	nad4L	303	1	303	1	303	1
	nad5	2010	5	2010	5	2010	5
	nad6	618	1	618	1	618	1
	nad7	1185	5	1185	5	1185	5
	nad9	573	1	573	1	573	1
Ribosomal protein (large)	*rpl16*	540	1	540	1	540	1
	rpl2	1050	2	1074	2	1050	2
	rpl5	558	1	558	1	558	1
Ribosomal protein (small)	*rps12*	378	1	378	1	378	1
	rps3	1671	2	1671	2	1671	2
	rps4	1089	1	1089	1	1089	1
	rps7	447	1	447	1	447	1

**Ribosomal RNA**	*rrn18*	1935	–	1935	–	1935	–
	rrn26	3169	–	3169	–	3169	–
	rrn5	118	–	118	–	118	–
**Transfer RNA**	*trnC-GCA*	71	–	71	–	71	–
	trnD-GUC	74	–	74	–	74	–
	trnE-UUC	72	–	72	–	72	–
	trnfM-CAU	74	–	74	–	71	–
	trnfM-CAU_2	–	–	–	–	74	–
	trnG-GCC	72	–	72	–	72	–
	trnH-GUG	74	–	74	–	74	–
	trnI-CAU	74	–	74	–	74	–
	trnK-UUU	73	–	73	–	73	–
	trnK-UUU_2	73	–	73	–	73	–
	trnM-CAU	73	–	73	–	73	–
	trnN-GUU	72	–	72	–	72	–
	trnP-UGG	75	–	75	–	75	–
	trnP-UGG_2	78	–	–	–	–	–
	trnQ-UUG	72	–	72	–	72	–
	trnQ-UUG_2	72	–	72	–	–	–
	trnS-GCU	88	–	88	–	88	–
	trnS-GCU_2	88	–	88	–	88	–
	trnS-GGA	87	–	87	–	87	–
	trnS-UGA	87	–	87	–	87	–
	trnS-UGA_2	–	–	–	–	87	–
	trnW-CCA	74	–	74	–	74	–
	trnY-GUA	83	–	83	–	83	–
	trnY-GUA_2	75	–	75	–	75	–
	trnY-GUA_3	75	–	76	–	–	–
	trnY-GUA_4	67	–	–	–	–	–
	trnY-GUA_5	67	–	–	–	–	–

Table S3. RNA editing analysis results.

Gene name – N sites	Mitogenome position	Gene position	AA position	Ref->Alt	RefCodon ->AltCodon	RefAA ->AltAA
***atp1* - 5 sites**	42927	15	5	C->T	CCC->CCT	P->P
	41764	1178	393	C->T	TCA->TTA	S->L
	41650	1292	431	C->T	CCG->CTG	P->L
	41527	1415	472	C->T	CCA->CTA	P->L
	41458	1484	495	C->T	CCA->CTA	P->L
***atp4* - 7 sites**	151478	89	30	C->T	TCA->TTA	S->L
	151429	138	46	C->T	ATC->ATT	I->I
	151352	215	72	C->T	TCG->TTG	S->L
	151319	248	83	C->T	CCT->CTT	P->L
	151316	251	84	C->T	CCG->CTG	P->L
	151172	395	132	C->T	TCA->TTA	S->L
	151151	416	139	C->T	ACT->ATT	T->I
***atp6* - 2 sites**	174597	287	96	C->T	TCC->TTC	S->F
	174755	445	149	C->T	CCA->TCA	P->S
***atp9* - 4 sites**	167602	20	7	C->T	TCA->TTA	S->L
	167572	50	17	C->T	TCA->TTA	S->L
	167488	134	45	C->T	TCA->TTA	S->L
	167431	191	64	C->T	CCA->CTA	P->L
***ccmB* – 30 sites**	160042	16	6	C->T	CTT->TTT	L->F
	160054	28	10	C->T	CAT->TAT	H->Y
	160097	71	24	C->T	TCA->TTA	S->L
	160106	80	27	C->T	TCG->TTG	S->L
	160154	128	43	C->T	TCA->TTA	S->L
	160163	137	46	C->T	TCC->TTC	S->F
	160174	148	50	C->T	CCG->TTG	P->L
	160175	149		C->T		
	160180	154	52	C->T	CGG->TGG	R->W
	160185	159	53	C->T	ATC->ATT	I->I
	160186	160	54	C->T	CCT->TCT	P->S
	160190	164	55	C->T	CCG->CTG	P->L
	160198	172	58	C->T	CCT->TCT	P->S
	160205	179	60	C->T	CCT->CTT	P->L
	160207	181	61	C->T	CCT->TCT	P->S
	160219	193	65	C->T	CCT->TTT	P->F
	160220	194		C->T		
	160312	286	96	C->T	CGG->TGG	R->W
	160330	304	102	C->T	CGT->TGT	R->C
	160364	338	113	C->T	TCG->TTG	S->L
	160393	367	123	C->T	CGG->TGG	R->W
	160405	379	127	C->T	CCA->TTA	P->L
	160406	380		C->T		
	160432	406	136	C->T	CTG->TTG	L->L
	160450	424	142	C->T	CGT->TGT	R->C

	160454	428	143	C->T	TCG->TTG	S->L
	160493	467	156	C->T	TCG->TTG	S->L
	160501	475	159	C->T	CCA->TTA	P->L
	160502	476		C->T		
	160511	485	162	C->T	TCA->TTA	S->L
***ccmC* - 28 sites**	144297	103	35	C->T	CAT->TAT	H->Y
	144267	133	45	C->T	CTT->TTT	L->F
	144221	179	60	C->T	GCG->GTG	A->V
	144216	184	62	C->T	CGG->TGG	R->W
	144069	331	111	C->T	CGG->TGG	R->W
	144005	395	132	C->T	TCG->TTG	S->L
	144000	400	134	C->T	CTT->TTT	L->F
	143979	421	141	C->T	CGT->TGT	R->C
	143964	436	146	C->T	CCT->TCT	P->S
	143954	446	149	C->T	CCG->CTG	P->L
	143942	458	153	C->T	TCA->TTA	S->L
	143937	463	155	C->T	CGT->TGT	R->C
	143933	467	156	C->T	GCT->GTT	A->V
	143927	473	158	C->T	CCG->CTG	P->L
	143903	497	166	C->T	TCT->TTT	S->F
	143879	521	174	C->T	TCG->TTG	S->L
	143852	548	183	C->T	TCT->TTT	S->F
	143832	568	190	C->T	CCT->TCT	P->S
	143825	575	192	C->T	CCC->CTC	P->L
	143792	608	203	C->T	CCC->CTC	P->L
	143786	614	205	C->T	TCA->TTA	S->L
	143782	618	206	C->T	ACC->ACT	T->T
	143781	619	207	C->T	CGT->TGT	R->C
	143776	624	208	C->T	ATC->ATT	I->I
	143750	650	217	C->T	CCT->CTT	P->L
	143745	655	219	C->T	CCA->TTA	P->L
	143744	656		C->T		
	143727	673	225	C->T	CCT->TCT	P->S
***ccmFC* - 12 sites**	85230	50	17	C->T	CCT->CTT	P->L
	85283	103	35	C->T	CCC->TCC	P->S
	85326	146	49	C->T	CCT->CTT	P->L
	85335	155	52	C->T	TCA->TTA	S->L
	85340	160	54	C->T	CCT->TCT	P->S
	85514	334	112	C->T	CGT->TGT	R->C
	85595	415	139	C->T	CTC->TTC	L->F
	87285	1150	384	C->T	CTA->TTA	L->L
	87307	1172	391	C->T	TCG->TTG	S->L
	87381	1246	416	C->T	CGG->TGG	R->W
	87415	1280	427	C->T	TCG->TTG	S->L
	87462	1327	443	C->T	CGA->TGA*	R->*
***ccmFN1* - 11 sites**	139860	38	13	C->T	CCG->CTG	P->L
	139800	98	33	C->T	CCT->CTT	P->L
	139761	137	46	C->T	TCG->TTG	S->L
	139747	151	51	C->T	CCT->TCT	P->S
	139642	256	86	C->T	CGG->TGG	R->W

	139635	263	88	C->T	CCA->CTA	P->L
	139615	283	95	C->T	CTT->TTT	L->F
	139125	773	258	C->T	TCA->TTA	S->L
	139113	785	262	C->T	CCA->CTA	P->L
	139098	800	267	C->T	TCA->TTA	S->L
	138949	949	317	C->T	CGC->TGC	R->C
***ccmFN2* - 10 sites**	59633	1184	395	C->T	TCG->TTG	S->L
	59744	1295	432	C->T	CCA->CTA	P->L
	59776	1327	443	C->T	CGG->TGG	R->W
	59794	1345	449	C->T	CGG->TGG	R->W
	59827	1378	460	C->T	CGG->TGG	R->W
	59845	1396	466	C->T	CGT->TGT	R->C
	59888	1439	480	C->T	TCG->TTG	S->L
	59912	1463	488	C->T	CCA->CTA	P->L
	59924	1475	492	C->T	TCA->TTA	S->L
	59959	1510	504	C->T	CCC->TCC	P->S
***cob* - 7 sites**	238768	286	96	C->T	CTT->TTT	L->F
	238807	325	109	C->T	CAT->TAT	H->Y
	239050	568	190	C->T	CAT->TAT	H->Y
	239335	853	285	C->T	CAT->TAT	H->Y
	239390	908	303	C->T	CCA->CTA	P->L
	239464	982	328	C->T	CAC->TAC	H->Y
	239566	1084	362	C->T	CCT->TCT	P->S
***cox2* - 13 sites**	252133	25	9	C->T	CTC->TTT	L->F
	252131	27		C->T		
	252087	71	24	C->T	TCT->TTT	S->F
	252020	138	46	C->T	CTC->CTT	L->L
	251905	253	85	C->T	CGG->TGG	R->W
	251880	278	93	C->T	TCG->TTG	S->L
	251779	379	127	C->T	CGG->TGG	R->W
	251682	476	159	C->T	TCA->TTA	S->L
	251601	557	186	C->T	CCT->CTT	P->L
	251577	581	194	C->T	TCA->TTA	S->L
	251460	698	233	C->T	ACG->ATG	T->M
	250097	721	241	C->T	CCT->TCT	P->S
	250076	742	248	C->T	CGG->TGG	R->W
***cox3* - 7 sites**	107483	112	38	C->T	CCA->TCA	P->S
	107616	245	82	C->T	CCT->CTT	P->L
	107628	257	86	C->T	TCT->TTT	S->F
	107682	311	104	C->T	TCT->TTT	S->F
	107685	314	105	C->T	TCT->TTT	S->F
	107784	413	138	C->T	CCT->CTT	P->L
	107793	422	141	C->T	CCT->CTT	P->L
***matR* - 9 sites**	34422	32	11	C->T	TCC->TTC	S->F
	34128	326	109	C->T	CCA->CTA	P->L
	34041	413	138	C->T	TCG->TTG	S->L
	32772	1682	561	C->T	TCC->TTC	S->F
	32751	1703	568	C->T	CCT->CTT	P->L
	32717	1737	579	C->T	TAC->TAT	Y->Y
	32695	1759	587	C->T	CAC->TAC	H->Y
	32607	1847	616	C->T	TCA->TTA	S->L

	32576	1878	626	C->T	ATC->ATT	I->I
***mttB* - 25 sites**	268478	49	17	C->T	CAT->TAT	H->Y
	268468	59	20	C->T	TCG->TTG	S->L
	268430	97	33	C->T	CGG->TGG	R->W
	268383	144	48	C->T	TTC->TTT	F->F
	268382	145	49	C->T	CCG->TCG	P->S
	268366	161	54	C->T	TCT->TTT	S->F
	268363	164	55	C->T	CCA->CTA	P->L
	268354	173	58	C->T	TCA->TTA	S->L
	268166	361	121	C->T	CTC->TTC	L->F
	268163	364	122	C->T	CAT->TAT	H->Y
	268148	379	127	C->T	CGC->TGC	R->C
	268118	409	137	C->T	CCC->TCC	P->S
	268087	440	147	C->T	CCA->CTA	P->L
	268022	505	169	C->T	CAT->TAT	H->Y
	267997	530	177	C->T	TCG->TTG	S->L
	267989	538	180	C->T	CCA->TCA	P->S
	267946	581	194	C->T	CCA->CTA	P->L
	267940	587	196	C->T	CCA->CTA	P->L
	267884	643	215	C->T	CCA->TCA	P->S
	267878	649	217	C->T	CTC->TTC	L->F
	267862	665	222	C->T	TCC->TTC	S->F
	267834	693	231	C->T	ATC->ATT	I->I
	267827	700	234	C->T	CGT->TGT	R->C
	267822	705	235	C->T	TTC->TTT	F->F
	267781	746	249	C->T	TCG->TTG	S->L
***nad1* - 21 sites**	286267	2	1	C->T	ACG->ATG*	T->M
	286102	167	56	C->T	TCG->TTG	S->L
	286004	265	89	C->T	CGG->TGG	R->W
	285962	307	103	C->T	CCG->TTG	P->L
	285961	308		C->T		
	285893	376	126	C->T	CGG->TGG	R->W
	127662	490	164	C->T	CCC->TCC	P->S
	127659	493	165	C->T	CGT->TGT	R->C
	127652	500	167	C->T	TCG->TTG	S->L
	127616	536	179	C->T	TCT->TTT	S->F
	127581	571	191	C->T	CTT->TTT	L->F
	127572	580	194	C->T	CGT->TGT	R->C
	127517	635	212	C->T	TCA->TTA	S->L
	31666	725	242	C->T	CCA->CTA	P->L
	31651	740	247	C->T	TCT->TTT	S->F
	31648	743	248	C->T	CCA->CTA	P->L
	31636	755	252	C->T	CCG->CTG	P->L
	31568	823	275	C->T	CTC->TTC	L->F
	31493	898	300	C->T	CGG->TGG	R->W
	31463	928	310	C->T	CGG->TGG	R->W
	31454	937	313	C->T	CCT->TCT	P->S
***nad2* - 23 sites**	10870	26	9	C->T	TCC->TTC	S->F
	10840	56	19	C->T	TCC->TTT	S->F
	10839	57		C->T		
	9664	252	84	C->T	TTC->TTT	F->F

	9608	308	103	C->T	TCT->TTT	S->F
	9605	311	104	C->T	TCC->TTC	S->F
	9522	394	132	C->T	CAT->TAT	H->Y
	9488	428	143	C->T	CCT->CTT	P->L
	230209	662	221	C->T	TCT->TTT	S->F
	232919	788	263	C->T	TCT->TTT	S->F
	232940	809	270	C->T	TCT->TTT	S->F
	233051	920	307	C->T	CCT->CTT	P->L
	233059	928	310	C->T	CAT->TAT	H->Y
	233089	958	320	C->T	CGT->TGT	R->C
	233093	962	321	C->T	ACT->ATT	T->I
	233189	1058	353	C->T	TCA->TTA	S->L
	233258	1127	376	C->T	TCG->TTG	S->L
	233377	1246	416	C->T	CCA->TTA	P->L
	233378	1247		C->T		
	233407	1276	426	C->T	CGT->TGT	R->C
	235194	1400	467	C->T	TCA->TTA	S->L
	235197	1403	468	C->T	TCC->TTC	S->F
	235251	1457	486	C->T	TCA->TTA	S->L
nad3 **- 11 sites**	56030	5	2	C->T	TCA->TTA	S->L
	56048	23	8	C->T	TCT->TTT	S->F
	56086	61	21	C->T	CTA->TTA	L->L
	56105	80	27	C->T	CCA->CTA	P->L
	56171	146	49	C->T	TCC->TTC	S->F
	56233	208	70	C->T	CCT->TTT	P->F
	56234	209		C->T		
	56272	247	83	C->T	CCT->TCT	P->S
	56276	251	84	C->T	CCC->CTC	P->L
	56369	344	115	C->T	TCG->TTG	S->L
	56374	349	117	C->T	CGG->TGG	R->W
nad4 **- 28 sites**	265612	29	10	C->T	TCT->TTT	S->F
	265567	74	25	C->T	ACT->ATT	T->I
	265534	107	36	C->T	CCG->CTG	P->L
	265517	124	42	C->T	CTG->TTG	L->L
	265483	158	53	C->T	CCT->CTT	P->L
	265477	164	55	C->T	CCT->CTT	P->L
	265475	166	56	C->T	CGG->TGG	R->W
	265444	197	66	C->T	TCT->TTT	S->F
	265324	317	106	C->T	TCA->TTA	S->L
	265279	362	121	C->T	ACA->ATA	T->I
	265265	376	126	C->T	CGT->TGT	R->C
	265238	403	135	C->T	CGC->TGC	R->C
	265205	436	146	C->T	CCC->TTC	P->F
	265204	437		C->T		
	265192	449	150	C->T	CCA->CTA	P->L
	263563	608	203	C->T	TCA->TTA	S->L
	263512	659	220	C->T	TCT->TTT	S->F
	263404	767	256	C->T	CCT->CTT	P->L
	260112	1010	337	C->T	CCG->CTG	P->L
	260089	1033	345	C->T	CCT->TCT	P->S
	260021	1101	367	C->T	TAC->TAT	Y->Y

	259993	1129	377	C->T	CTC->TTC	L->F
	259974	1148	383	C->T	TCT->TTT	S->F
	259950	1172	391	C->T	TCA->TTA	S->L
	259767	1355	452	C->T	TCA->TTA	S->L
	259749	1373	458	C->T	TCC->TTC	S->F
	257769	1417	473	C->T	CAC->TAC	H->Y
	257753	1433	478	C->T	CCG->CTG	P->L
***nad4L* - 9 sites**	152099	41	14	C->T	TCT->TTT	S->F
	152085	55	19	C->T	CGG->TGG	R->W
	152054	86	29	C->T	CCT->CTT	P->L
	152045	95	32	C->T	TCA->TTA	S->L
	152030	110	37	C->T	TCA->TTA	S->L
	152009	131	44	C->T	TCG->TTG	S->L
	151982	158	53	C->T	TCG->TTG	S->L
	151952	188	63	C->T	TCA->TTA	S->L
	151943	197	66	C->T	CCA->CTA	P->L
***nad5* - 29 sites**	31046	155	52	C->T	CCG->CTG	P->L
	30130	242	81	C->T	CCG->CTG	P->L
	30100	272	91	C->T	TCC->TTC	S->F
	30014	358	120	C->T	CTT->TTT	L->F
	29998	374	125	C->T	TCA->TTA	S->L
	29974	398	133	C->T	TCT->TTT	S->F
	29878	494	165	C->T	ACA->ATA	T->I
	29824	548	183	C->T	TCG->TTG	S->L
	29819	553	185	C->T	CGT->TGT	R->C
	29774	598	200	C->T	CGT->TGT	R->C
	29763	609	203	C->T	GCC->GTT	A->V
	29764	608		C->T		
	29743	629	210	C->T	TCT->TTT	S->F
	29696	676	226	C->T	CTT->TTT	L->F
	29659	713	238	C->T	TCG->TTG	S->L
	29647	725	242	C->T	TCA->TTA	S->L
	29608	764	255	C->T	TCG->TTG	S->L
	29537	835	279	C->T	CCA->TCA	P->S
	29509	863	288	C->T	TCT->TTT	S->F
	29497	875	292	C->T	ACG->ATG	T->M
	28972	1400	467	C->T	TCA->TTA	S->L
	77870	1490	497	C->T	CCC->CTC	P->L
	77810	1550	517	C->T	ACC->ATC	T->I
	77780	1580	527	C->T	TCA->TTA	S->L
	77750	1610	537	C->T	CCC->CTC	P->L
	76487	1895	632	C->T	TCA->TTA	S->L
	76466	1916	639	C->T	TCT->TTT	S->F
	76464	1918	640	C->T	CGT->TGT	R->C
	76424	1958	653	C->T	TCG->TTG	S->L
***nad6* - 11 sites**	6864	26	9	C->T	CCT->CTT	P->L
	6837	53	18	C->T	GCA->GTA	A->V
	6802	88	30	C->T	CCC->TTC	P->F
	6801	89		C->T		
	6795	95	32	C->T	CCA->CTA	P->L
	6787	103	35	C->T	CGC->TGC	R->C

	6729	161	54	C->T	CCA->CTA	P->L
	6721	169	57	C->T	CAT->TAT	H->Y
	6699	191	64	C->T	TCA->TTA	S->L
	6444	446	149	C->T	TCT->TTT	S->F
	6427	463	155	C->T	CCT->TCT	P->S
***nad7* - 19 sites**	20246	24	8	C->T	ATC->ATT	I->I
	20260	38	13	C->T	TCG->TTG	S->L
	20299	77	26	C->T	TCA->TTA	S->L
	20359	137	46	C->T	TCA->TTA	S->L
	21385	200	67	C->T	TCT->TTT	S->F
	21394	209	70	C->T	TCA->TTA	S->L
	22496	244	82	C->T	CAT->TAT	H->Y
	22503	251	84	C->T	TCA->TTA	S->L
	22568	316	106	C->T	CGT->TGT	R->C
	22587	335	112	C->T	TCA->TTA	S->L
	22596	344	115	C->T	TCA->TTA	S->L
	22830	578	193	C->T	TCA->TTA	S->L
	24003	698	233	C->T	TCG->TTG	S->L
	24029	724	242	C->T	CAT->TAT	H->Y
	24039	734	245	C->T	TCG->TTG	S->L
	24044	739	247	C->T	CTT->TTT	L->F
	24074	769	257	C->T	CGC->TGC	R->C
	26036	926	309	C->T	TCA->TTA	S->L
	26167	1057	353	C->T	CGT->TGT	R->C
***nad9* - 7 sites**	79942	92	31	C->T	TCT->TTT	S->F
	79867	167	56	C->T	TCG->TTG	S->L
	79844	190	64	C->T	CAT->TAT	H->Y
	79736	298	100	C->T	CCG->TCG	P->S
	79706	328	110	C->T	CGG->TGG	R->W
	79636	398	133	C->T	TCA->TTA	S->L
	79595	439	147	C->T	CTT->TTT	L->F
***rpl16* - 6 sites**	155147	30	10	C->T	CTC->CTT	L->L
	155116	61	21	C->T	CAG->TAG*	Q->*
	154968	209	70	C->T	ACT->ATT	T->I
	154737	440	147	C->T	CCA->CTA	P->L
	154671	506	169	C->T	CCA->CTA	P->L
	154665	512	171	C->T	TCG->TTG	S->L
***rpl5* - 11 sites**	236181	47	16	C->T	CCG->CTG	P->L
	271173	212	71	C->T	CCA->CTA	P->L
	236169	35	12	C->T	TCA->TTA	S->L
	236192	58	20	C->T	CCG->TTG	P->L
	236193	59		C->T		
	236198	64	22	C->T	CAC->TAC	H->Y
	236226	92	31	C->T	TCG->TTG	S->L
	236303	169	57	C->T	CGC->TGC	R->C
	236451	317	106	C->T	TCG->TTG	S->L
	236463	329	110	C->T	TCG->TTG	S->L
	236646	512	171	C->T	CCA->CTA	P->L
***rps12* - 7 sites**	56511	84	28	C->T	CCC->CCT	P->P
	56531	104	35	C->T	CCG->CTG	P->L
	56573	146	49	C->T	CCA->CTA	P->L

	56623	196	66	C->T	CAC->TAC	H->Y
	56648	221	74	C->T	TCG->TTG	S->L
	56696	269	90	C->T	TCG->TTG	S->L
	56711	284	95	C->T	TCC->TTC	S->F
***rps3* - 8 sites**	158234	64	22	C->T	CGG->TGG	R->W
	156111	603	201	C->T	TTC->TTT	F->F
	155370	1344	448	C->T	AGC->AGT	S->S
	155362	1352	451	C->T	CCG->CTG	P->L
	155244	1470	490	C->T	TCC->TCT	S->S
	155180	1534	512	C->T	CGT->TGT	R->C
	155147	1567	523	C->T	CCT->TCT	P->S
	155116	1598	533	C->T	TCA->TTA	S->L
***rps4* - 16 sites**	12673	77	26	C->T	TCA->TTA	S->L
	12662	88	30	C->T	CGG->TGG	R->W
	12575	175	59	C->T	CCG->TCG	P->S
	12524	226	76	C->T	CCC->TCC	P->S
	12451	299	100	C->T	CCA->CTA	P->L
	12442	308	103	C->T	CCA->CTA	P->L
	12418	332	111	C->T	CCG->CTG	P->L
	12373	377	126	C->T	CCG->CTG	P->L
	12226	524	175	C->T	TCA->TTA	S->L
	11794	956	319	C->T	TCG->TTG	S->L
	11758	992	331	C->T	TCT->TTT	S->F
	11783	967	323	C->T	CAT->TAT	H->Y
	11708	1042	348	C->T	CCA->TTA	P->L
	11707	1043		C->T		
	11698	1052	351	C->T	CCT->CTT	P->L
	11693	1057	353	C->T	CGG->TGG	R->W
rps7	282833	332	111	C->T	TCA->TTA	S->L

* - start/stop codon editing
